# *Candida parapsilosis* as a Causative Agent of Onychomycosis in Patient with Cirrhosis of the Liver

**DOI:** 10.3390/jof6040313

**Published:** 2020-11-25

**Authors:** Yelena Kukhar, Ainura Smagulova, Ainash Daniyarova, Aliya Baiduissenova, Vladimir Kiyan

**Affiliations:** 1 Department of Microbiology and Biotechnology, S. Seifullin Kazakh Agrotechnical University, Nur-Sultan 010000, Kazakhstan; kukharelena1808@gmail.com (Y.K.); a.daniyarova01@gmail.com (A.D.); 2 Research platform of Agricultural Biotechnology, S. Seifullin Kazakh Agrotechnical University, Nur-Sultan 010000, Kazakhstan; smagulova0114@gmail.com; 3 Department of Microbiology, Virology and Immunology, Astana Medical University, Nur-Sultan 010000, Kazakhstan; alyia6512@gmail.com

**Keywords:** onychomycosis, candidiasis, *Candida parapsilosis*, yeast

## Abstract

We report the first case of non-dermatophytic onychomycosis of the toenail described in Kazakhstan caused by *Candida parapsilosis*. The biological properties of the strain were studied. *C. parapsilosis* forms white creamy colonies, smooth with focal wrinkles, and the reversum is light-yellow. The culture of *C. parapsilosis* is represented by a yeast form, characterized by the presence of round or cylindrical yeast cells with active budding. The strain has a high saccharolytic and urease activity and is indifferent to the sucrose and maltose. The *C. parapsilosis* strain was sensitive to polyene and azole antifungal agents. The highest sensitivity was found to ketoconazole, itraconazole and nystatin.

## 1. Introduction

Onychomycosis is an infection of the nail plate and bed, which is caused by three groups of fungal pathogens: dermatophyte molds (DM), non-DM (NDM) and yeasts [1,2]. The spectrum of yeast causing onychomycosis of the feet and hands includes more than nine species of the genus *Candida* spp., *Trichosporon* spp., *Malassezia* spp. and other genera [3]. Over the years, members of the genus *Candida* considered saprophytic commensals occur most commonly on the skin and mucous membranes of humans. However, it is reported that some species belonging to the genus *Candida* (*C. albicans*, *C. tropicalis*, *C. parapsilosis*, *C. glabrata*) are pathogenic because they cause pathological processes known as candidiasis and moniliasis. In the etiology of onychomycosis of the feet, *C. albicans* occupies an average of 0.7–8.3%. Onychomycosis of the hands is, in 44.5% of cases, caused by *Candida* spp. [4]. Yeasts, particularly *C. albicans*, are mainly isolated from fingernails in chronic paronychia and onycholysis, and from nails in chronic mucocutaneous candidosis [5].

Until recently, it was believed that *C. albicans* is the most common pathogen isolated from the affected nails and toes. As the most common pathogens, *C. albicans* are found in India (24.27%), Israel (19.5–34.4%) and Iran (84%) [6,7,8].

Currently, *C. parapsilosis* is becoming one of the important etiological agents of opportunistic onychomycosis, especially in patients with immunosuppression [2,9]. This study describes onychomycosis cases where *C. parapsilosis* occurs as a complex with other fungal pathogens [7,10] and as the sole causative agent of onychomycosis [11,12].

It has been suggested that *C. parapsilosis* may be the most common *Candida* species for infections of the fingers and toes, because the isolation of *C. parapsilosis* is more common than *C. guillermondii* and *C. albicans* in onychomycosis. Thus, when analyzing the fingernail and toenail samples, the authors revealed that *C. parapsilosis* was the most commonly isolated strain with 43.3% cases, while isolates of *C. guillermondii* and *Candida albicans* were 24.2% and 23.6%, respectively [13]. It is shown that *C. parapsilosis* was leading yeast pathogen that infects fingernails (50%) and toenails (39%), and the second most common agent of onychomycosis (12%) after the dermatophyte *Trichophyton rubrum* [14].

For the first time in the Republic of Kazakhstan, we have established a case of onychomycosis caused by the opportunistic invasive pathogen *C. parapsilosis* isolated from pathological material. The basic biological properties of the opportunistic pathogen of onychomycosis were studied and its species affiliation was established.

## 2. Case Report and Results

In the case of a 25-year-old male patient of Asian descent, with a tendency to be overweight, during primary sanitization, an extensive lesion of all the toenails of both feet was revealed in the form of clouding and thickening, as well as the accumulation of mucous mass in the subungual and interdigital spaces; the fingernails of both hands were without visible changes with slight redness of the periungual roller. Toenails were damaged in the form of deformation and a dull dirty-gray color with an uneven, thickened, bumpy surface. The toenail roller is inflamed, pillow-shaped overhanging over the nail, with the presence of foci of peeling along the edge. In the interdigital spaces of the foot, an accumulation of mucus-like mass with an unpleasant odor is accompanied by itching. The presence of a specific odor from the patient’s body was noted, body mass index 28.3. The patient works in a confectionery shop, abuses sweets, has never been treated for mycosis of the feet or onychomycosis.

Scraping from the edges, the outer and inner surfaces of the proximal and lateral periungual ridges, as well as nail clippings from different parts of the affected toenail, were collected on day 0 after proper sterilization of the affected area with 70% alcohol and transported to the mycology research laboratory, Microbiology and Biotechnology Department at S. Seifullin Kazakh Agrotechnical University, for examination by culture method.

During the initial isolation of the pathogen (1 day) and upon obtaining a pure culture, the surface cultivation of the fungus was carried out at a temperature of 32 °C for at least 6 day until the formation of characteristic colonies. Mycological diagnosis was performed at +10 days. The colonies are smooth, moist, and creamy. On a liquid nutrient medium, cultures of yeast-like fungi grew in the form of a low parietal layer and precipitate. Filamentation on potato agar—Pseudomycelia—is poorly developed, deprived of chlamydospores and has a very low glycolytic activity.

**Figure 1:**
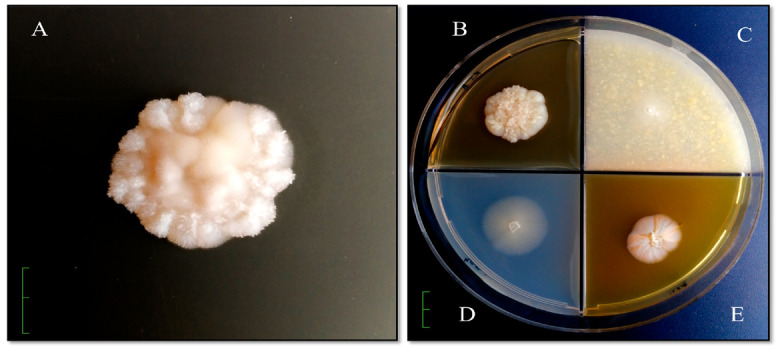
Macroscopic appearance of cultured colony of *C. parapsilosis* №398.2 on Sabouraud agar (**A**), potato agar (**B**), corn agar (**C**), Chapek’s medium (**D**) and honey agar (**E**), 32 °C, 6 days, bar = 1 cm.

To study the cultural and morphological properties of the isolated *Candida* yeast strain, agar media were used (Sabouraud’s medium, Chapek’s medium, and media based on honey and corn) (Figure 1). Phenotypic identification was performed using the determinant of microorganisms [15].

Colonies grow rapidly on Sabouraud agar, initially forming smooth, shiny, unevenly rounded, creamy colonies with a diameter of 2.5 cm × 2.3 cm. The front side of the colonies in a pure culture is initially smooth, even, uniform, round. From the front, colonies in a pure culture are initially smooth, even, uniform, round. As the colonies grow, they become folded and striated, become thick, greasy, hilly, and as the biomass accumulates and the colony thickens, they crack in the center. Over time, from the reverse, they are milk cream color (Figure 1A). On potato agar, colonies with a diameter of 4.1 cm × 3.5 cm are formed, wrinkled with pronounced zonality, intensely wrinkled in the center, with a pronounced growing edge (Figure 1B). Colonies on corn agar are uniformly round, hilly in the center, diameter 1.5 × 1.3 cm, milky white, and the reversum is not stained (Figure 1C). From the front and reverse, on Chapek’s medium are formed flat, translucent, colorless colonies with a raised center with a diameter of 2.2 cm × 2.2 cm (Figure 1D). Colonies on honey agar are shiny, round, wrinkled in the center, pigmented from the back to brown and intense pigment release into the nutrient substrate, diameter 1.9 cm × 1.5 cm (Figure 1E).

The morphological feature of this strain on Sabouraud agar, potato and honey agar is the fine-needle surface of the colonies. An imitation of a fluffy colony was revealed on Chapek’s medium, and a smooth surface on corn agar.

**Figure 2:**
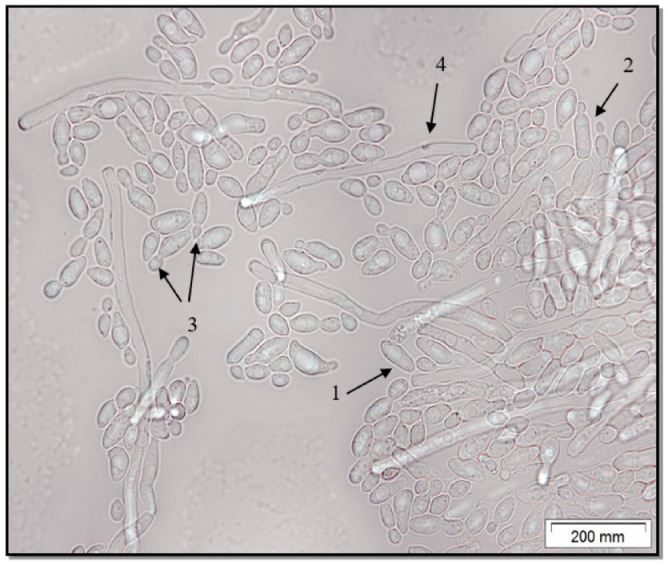
Microscopic structures of *C. parapsilosis* such as the yeast form are oval (**1**) and cylindrical (**2**); budding stage (**3**); pseudohyphae (**4**).

Microscopic analysis of the *C. parapsilosis* culture showed that it is represented by a yeast form (Figure 2). The *C. parapsilosis* strain is characterized by the presence in the field of view of round or cylindrical yeast cells, including budding. A small amount of pseudohyphae is noted.

Genomic DNA was extracted from *C. parapsilosis* cells using the liquid nitrogen and phenol-chloroform extraction method, and the genomic DNA was analyzed by electrophoresis on 1% agarose gel. The *ITS* region on rDNA was amplified by using specific primers *ITS4* (5′-TCCTCCGCTTATTGATATGC-3′) and *ITS5* (5′-GGAAGTAAAAGTCGTAACAAGG-3′) (Integrated DNA Technologies, Inc., Coralville, IA, USA) and received the PCR product with a size of 526 bp. The PCR reaction was done in a SimpliAmp thermal cycler (Applied biosystems, Waltham, MA, USA) under the following conditions: an initial denaturation set up at 94 °C for 5 min was followed by 35 cycles of denaturation at 95 °C for 30 s, annealing at 52 °C for 40 s and extension at 72 °C for 50 s, with a final extension step of 72 °C for 7 min. The sequencing was done by using BigDye^®^ Terminator v3.1 Cycle Sequencing Kit (Applied Biosystems) and the sequence was deposited in GenBank with accession no. MT482736.1 (*ITS*). These sequences were compared with other sequences in the GenBank by using the BLAST analysis. The phylogenetic analysis was carried out with MEGA 6 software (The Pennsylvania State University, University Park, PA, USA).

**Figure 3:**
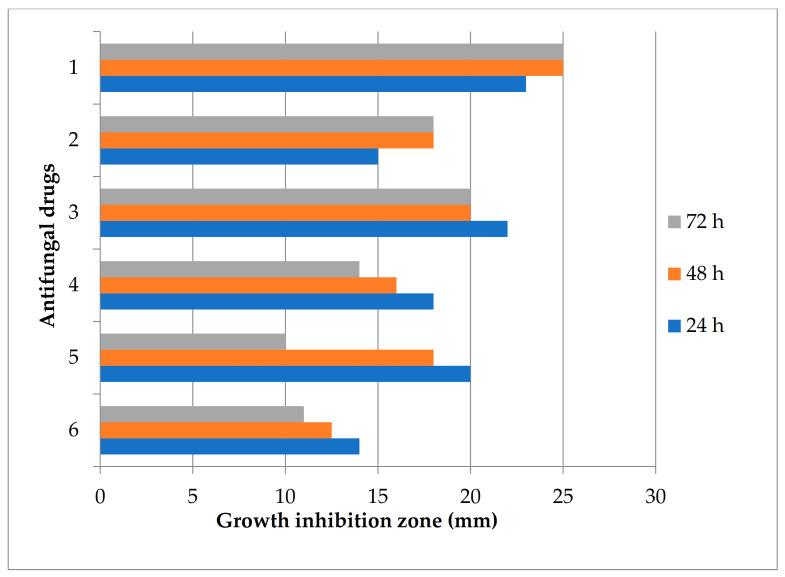
Analysis of the sensitivity of drugs to the causative agent of *C. parapsilosis*: (1) Ketoconazole, 20 mcg; (2) Itraconazole, 10 mg; (3) Fluconazole, 40 mcg; (4) Clotrimazole, 10 mcg; (5) Nystatin, 80 units; (6) Amphotericin B, 40 mg.

Determination of the sensitivity of *C. parapsilosis* was carried out by the disk-diffusion method with respect to the main groups of antifungal drugs polyene (amphotericin B and nystatin) and azole antifungal agents (clotrimazole, fluconazole, ketoconazole, itraconazole). The value of the presence of zones of growth inhibition of the colony to antifungal drugs: stable (0–6 mm), slightly sensitive (7–15 mm), sensitive (16–24 mm) and highly sensitive (25 and more mm). Experimental data show that nystatin, fluconazole and ketoconazole have a reliable fungicidal effect on the first day (Figure 3).

By the third day, the activity of nystatin against *C. parapsilosis* decreases sharply, as does the effect of amphotericin, clotrimazole and fluconazole. Against this background, the effect of itraconazole and ketoconazole is clearly visible, the effect of which increases with the accumulation of the antifungal drug in the agar.

At +14 days, complex therapy with systemic and local antimycotics was prescribed: oral ketoconazole (200 mg/day) in combination with clotrimazole cream (1%), twice per day were given for 1 month. A change in diet is recommended: the complete exclusion of sweets and the inclusion of fish, fresh vegetables and fruits in the diet. At the next appointment, the patient complained of liver enlargement, colic, and vomiting. An ultrasound scan revealed cirrhosis of the liver. In the future, the patient was not observed in the dermatological clinic due to treatment in the oncology clinic; therefore, the patient’s condition cannot be confirmed.

## 3. Discussion

In the present study, we isolated a fungus strain from the toenail of a 25-year-old man that was assigned to the genus *Candida*. We confirmed the identification of our isolate to species level as *C. parapsilosis* (accession no. MT482736.1) by DNA sequence analysis. A nucleotide BLAST search with 243 bp showed a maximum homology of 99.42–100% with *C. parapsilosis* of Asian origin: Japanese—44%, Iranian—19%, Qatari—12%, Indian—8%, Chinese—7%, Iraqi—3%, Turkish—2%, Russian, Malaysian and Sri Lankan in 2% of cases, respectively.

At the same time, only 12 of the 100 homologous strains presented in the NCBI are isolated from clinical material obtained from humans. Of these 12 strains, only one was isolated from the biomaterial of the third left finger of a person (Malaysia, MN826321.1), and three strains (MN944467.1, MN515420.1, MN044944.1) are associated with yeast skin lesions (seborrhea, deep skin candidiasis, scalp infection). One of the strains was isolated from Kazakh soil (MH059651.1), which may explain the etiology of foot onychomycosis.

Candidiasis is one of the common fungal opportunistic infections associated with various types of *Candida*. Numerous studies show that, previously, *C. albicans* was the most frequently released yeast in onychomycosis. However, increasingly, the authors report that *C. parapsilosis* was the most frequently isolated fungus for onychomycosis of the toenails [7,10,16]. The authors explain the frequent registration of *C. glabrata*, *C. tropicalis*, and *C. parapsilosis* species as human pathogens by the improvement of identification methods and the introduction of molecular methods in the routine diagnosis of fungemia [14,16]. In fact, *C. parapsilosis* is increasingly referred to as the most common agent for candida onychomycosis [14]. This is proved by the data of cultural-morphological and molecular genetic studies of various authors [14,17]. It should be noted that opportunistic microorganisms can cause onychomycosis in patients with asymmetric gait nail unit syndrome [18].

It is known that *C. parapsilosis* cannot exist in several morphogenetic forms and does not form complete hyphae. Laffey S. et al. reported that the colony phenotype depends on the form of *C. parapsilosis*: yeast colonies exhibit smooth or crater phenotypes, while pseudohyphae exhibit crepe or concentric phenotypes [17].

Thus, the culture of *C. parapsilosis* that we isolated, which caused damage to the patient’s nail plate, was the only pathogen. The strain was distinguished by characteristic species features. On dextrose Sabouraud’s medium, smooth colonies were observed acquiring a crater appearance and wrinkling as biomass accumulated; this is characteristic of the yeast phase of *C. parapsilosis* (Figure 1). This was confirmed by light microscopy, where it is clearly visible that the cells of *C. parapsilosis* are oval or cylindrical in shape (Figure 2).

Biochemical analysis made it possible to establish a high enzymatic activity of the strain: pronounced urease activity and the presence of enzymes that break down glucose and mannitol; low saccharolytic activity against sucrose, maltose and lactose. It was reported earlier that *C. parapsilosis* is not able to ferment sucrose and maltose [14]. The sensitivity of the identified culture in relation to five antimycotics was studied. This study reveals that the *C. parapsilosis* strain was sensitive to all drugs, but to varying degrees (Figure 3). The highest sensitivity was found to ketoconazole, itraconazole and nystatin.

In conclusion, we report the case of onychomycosis of a toenail of an inpatient with cirrhosis of the liver caused by *C. parapsilosis*. Identification of the causal agent was confirmed by molecular methods. Immunodeficiency and malignant neoplasm are likely to be a predisposing factor in our reported case.
